# Diseased Tendon Models Demonstrate Influence of Extracellular Matrix Alterations on Extracellular Vesicle Profile

**DOI:** 10.3390/bioengineering11101019

**Published:** 2024-10-12

**Authors:** Kariman A. Shama, Zachary Franklin Greenberg, Chadine Tammame, Mei He, Brittany L. Taylor

**Affiliations:** 1 J. Crayton Pruitt Family Department of Biomedical Engineering, University of Florida, Gainesville, FL 32611, USA; k.shama@ufl.edu (K.A.S.); tammame.chadine@ufl.edu (C.T.); mhe@cop.ufl.edu (M.H.); 2 Department of Pharmaceutics, University of Florida, Gainesville, FL 32603, USA; zfg2013@ufl.edu

**Keywords:** nanofibrous scaffolds, electrospinning, biomimicry, extracellular vesicles, tendon

## Abstract

Tendons enable movement through their highly aligned extracellular matrix (ECM), predominantly composed of collagen I. Tendinopathies disrupt the structural integrity of tendons by causing fragmentation of collagen fibers, disorganization of fiber bundles, and an increase in glycosaminoglycans and microvasculature, thereby driving the apparent biomechanical and regenerative capacity in patients. Moreover, the complex cellular communication within the tendon microenvironment ultimately dictates the fate between healthy and diseased tendon, wherein extracellular vesicles (EVs) may facilitate the tendon’s fate by transporting biomolecules within the tissue. In this study, we aimed to elucidate how the EV functionality is altered in the context of tendon microenvironments by using polycaprolactone (PCL) electrospun scaffolds mimicking healthy and pathological tendon matrices. Scaffolds were characterized for fiber alignment, mechanical properties, and cellular activity. EVs were isolated and analyzed for concentration, heterogeneity, and protein content. Our results show that our mimicked healthy tendon led to an increase in EV secretion and baseline metabolic activity over the mimicked diseased tendon, where reduced EV secretion and a significant increase in metabolic activity over 5 days were observed. These findings suggest that scaffold mechanics may influence EV functionality, offering insights into tendon homeostasis. Future research should further investigate how EV cargo affects the tendon’s microenvironment.

## 1. Introduction

Tendon is a connective tissue that transmits physical forces to bone to enable active movement [1]. While tendon composition varies by anatomic location, all tendons share a common feature of a highly aligned, anisotropic extracellular matrix (ECM) predominantly composed of collagen I, which provides tensile strength and resilience [2]. In healthy tendons, collagen fibers are highly organized, with various cells, predominantly tenocytes, aligned along the length of these fibers. The diameter of collagen fibers in healthy tendons typically ranges from 1 to 20 µm [3]. Upon the development of tendinopathy, the afflicted tendon begins losing regenerative capability, with the mechanisms underlying the tendon’s reduced regenerative capacity being poorly understood [4]. Diseased tendons exhibit fragmented collagen fibers, disorganized collagen bundles, an accumulation of glycosaminoglycans, and increased microvasculature associated with neoinnervation [5]. Subsequently, diseased tendon structure results in altered collagen fibril orientation and thickness [6], reducing the tendon functionality significantly. Specifically, the topographically altered tendon ECM leads to a decreased capacity to bear loads, partly due to a reduction in the diameter of type III collagen fibrils [7]. Furthermore, other reports have shown how diseased tendon’s ECM transitions from a state of hypovascularity and hypocellularity to being hypervascular and hypercellular [8], which instantiates a highly active cellular microenvironment and potential for necessary cell communication, which is yet to be fully elucidated in tendon pathology. Cells communicate with each other through a variety of mechanisms, with one method being through extracellular vesicles (EVs), serving as mediators of tissue remodeling and homeostasis [9]. Therefore, we believe that EVs within the tendon microenvironment are actively mediating tendinopathy development.

Derived from various cell types, EVs are membrane-bound vesicles that facilitate intercellular communication during tissue healing by transporting biomolecules among cells in the injured tissue microenvironment [10,11]. In recent years, EVs have garnered attention as a cell-free therapeutic and diagnostic biomarker in various pathologies, including cardiovascular and renal diseases, neurological disorders, fibrosis, and cancer [12,13,14,15]. In fibrotic diseases, EVs transport disease-specific cargo that contributes to the pathology of fibrosis [16]. For example, in lung fibrosis, EVs containing WNT5A can promote fibrotic remodeling of lung tissue [17,18]. Similarly, in renal fibrosis, EVs promote pathophysiology by transferring TGF-β mRNA through exosomes released by injured tubular epithelial cells [19]. EVs have been suggested to facilitate the progression of other pathologies, as cancer-derived EVs have been implicated in altering healthy fibroblasts to cancer-associated fibroblast phenotypes [20]. Furthermore, an increased number of EVs in human plasma has been suggested as a stand-alone diagnostic marker for cancer [21,22]. With the progression of disease, the stiffness of the tumor microenvironment increases, and a growing body of evidence suggests that a rigid extracellular matrix (ECM) can elevate EV secretion from tumor cells, which contributes to tumor growth [23,24]. EVs dynamically interact with ECM through various mechanisms [25,26]. Cancer-derived EVs suggest that specific molecules on their surface play a role in matrix remodeling and degradation, thereby advancing disease pathology. For example, heparanase remains on the surface of EVs upon their release, and when these EVs encounter the ECM, they facilitate the degradation of heparan sulfate components [27]. The presence of matrix-degrading molecules on EVs indicates their involvement in matrix degradation and potentially in the progression of pathological conditions. Yet, EV involvement in cell–cell communication in tendon pathology, immunomodulation, and healing is still not fully elucidated but could offer potential insights into the mechanisms underlying insufficient tendon recovery.

In vitro models are essential experimental tools for studying cellular interactions and understanding the mechanisms of specific pathologies within a controlled environment. Although animal models are considered the gold standard for these studies, in vitro models allow for greater control over experimental conditions, such as mechanical and topographical cues, while avoiding confounding factors like systemic inflammation that can be present in animal models [3,28]. Despite the vastly different mechanical properties among species, small animal models of tendinopathy fail to accurately reflect human tendon repair mechanisms [3,29,30]. Diseased tendon models can be generated by introducing specific stressors or topographical modifications to simulate tendinopathy conditions [31]. Furthermore, conditioned cell cultures can be employed for various translational applications, including the diagnosis and prognosis of different pathologies. Despite extensive research over the past decades on the healing mechanisms of tendons, the majority of cellular components and processes involved in tendon repair remain poorly elucidated. This underscores the necessity for developing advanced in vitro models to study the intricate multicellular interactions within tendon microenvironments [3]. Electrospinning allows the creation of highly aligned and fibrotic microenvironments that emulate the ECM of homeostatic and diseased tendons, respectively. Additionally, it permits precise tuning of mechanical and topographical cues. These in vitro models enable the study of EVs in both healthy and diseased states, providing insights into their potential mechanistic role in tendon pathology. To investigate the influence of the tendon healing microenvironments on EV biogenesis and profile, we created highly aligned and unaligned polycaprolactone (PCL) electrospun scaffolds with varying fiber densities. These scaffolds mimic the biophysical components of disorganized pathologic and healthy tendinous tissue, respectively. Synthetic ECM analogs, such as electrospun PCL, can effectively replicate the fibrous structure of native tendinous tissues. The presence of pores and fibrils within the ECM facilitates the passive transport of EVs through the interstitial space [25]. Numerous studies have shown that EVs accumulate in significant quantities within the interstitial space of the ECM [32,33,34]. Our goal is to characterize EVs within our in vitro tendon models by examining differences in EV concentration, heterogeneity, and cells across different microenvironments. Our hypothesis is that EV secretion profiles will correlate with the tissue microenvironment, with scaffolds emulating tendon pathology leading to increased EV production and elevated EV secretion per cell. Additionally, our hypothesis posits that our nanofibrous scaffolds that are more biomimetic of pathologic tendon will have more cell proliferation and enhanced metabolic activity.

## 2. Materials and Methods

### 2.1. Electrospinning

PCL (Thermo Scientific; Cat: 178305000; Fair Lawn, NJ, USA) solutions were prepared by dissolving PCL in dichloromethane (DCM) (Sigma-Aldrich; Cat: 270997; St. Louis, MO, USA) and dimethylformamide (DMF) (Thermo Scientific; Cat: 279600040; Fair Lawn, NJ, USA) overnight at room temperature to achieve 75% and 50% weight per volume (*w*/*v*) solutions. PCL was selected as the polymer of choice due to its attributes, including non-toxicity, biodegradability, slow degradation rate, robust mechanical properties, and biocompatibility [35]. Furthermore, its extensive utilization in tendon therapeutics and in vitro models further justified its selection [36]. These solutions were subsequently electrospun onto a rotating cylindrical aluminum mandrel at speeds of either 100 or 800 revolutions per minute (RPM), positioned approximately 13 cm away from the needle tip. Electrospinning was carried out under a high-voltage power supply generating a voltage potential of 26 kV. The polymer extrusion rate was maintained at 5 mL/h, with ambient conditions set to a relative humidity range of 35–46% and a temperature of 26 °C. These specific parameters were determined through systematic optimization of various polymer concentrations and flow rates (data not presented) as well as described previously [37].

### 2.2. Scaffold Characterization

Tensile mechanical testing was conducted on the scaffolds employing a ramp-to-failure protocol with a strain rate of 0.3% per second. The ultimate tensile strength and Young’s modulus were quantified and analyzed to assess the impact of scaffold mechanics on extracellular vesicle (EV) secretion. All nanofibrous scaffolds were added to a ZEISS/LEO SEM Pin Stub Mount, sputter coated with gold-palladium, and underwent scanning electron microscopy (SEM) utilizing the Phenom Pure Desktop SEM (Thermo Scientific; Waltham, MA, USA). All SEM micrographs underwent fibril alignment and diameter analysis using ImageJ 1.54f and its plugin OrientationJ.

### 2.3. Cell Culture

Nanofibrous scaffolds were trimmed to match the diameter of a 6-well tissue culture plate, sterilized, and then seeded with NIH3T3 fibroblasts at a density of 52,000 cells per square centimeter in high glucose DMEM supplemented with 10% exosome-depleted fetal bovine serum (Fisher; Cat: A2720801; Long Island, NY, USA) and 1% penicillin-streptomycin. Media was collected on Days 1, 3, and 5 for the isolation of EVs using a qEVoriginal 70 nm size exclusion column (Izon Science; Cat: ICO-70; Christchurch, New Zealand). Briefly, the conditioned media underwent low-speed centrifugation at 1500× *g* for 10 min followed by 10,000× *g* for 10 min to eliminate cells, cell debris, and apoptotic bodies. Subsequently, the supernatants were loaded on top of a qEV 70 nm size exclusion column and eluted according to the manufacturer’s instructions. Fractions containing EVs were collected for further downstream characterization.

### 2.4. EV Characterization

Nanoparticle tracking analysis (NTA) was conducted utilizing the Zetaview nanoparticle tracking analyzer (Particle Metrix; Ammersee, Germany) to ascertain the size distribution and concentration of EVs (*N* = 3 per group). EV samples were appropriately resuspended and diluted in cold PBS to achieve the optimal working concentration for the NTA system. Measurements were acquired at 11 distinct positions using a wavelength of 488 nm for each sample. EV protein content was measured (*N* = 3 per group) via Micro BCA™ Protein Assay Kit (Fisher, Cat: 23235). Analysis of EV-specific markers, TSG101, CD63, CD81, was conducted with an automatized capillary electrophoresis system (JESS, ProteinSimple; San Jose, CA, USA). Western blot band intensity was quantified using ImageJ 1.54f. Transmission electron microscopy (TEM) analysis was performed to assess EV morphology (Talos L120C; Thermo Scientific; Waltham, MA, USA).

### 2.5. Cellular Activity Assessment

To assess the metabolic activity of the cells cultured on the scaffolds, an MTS assay was conducted (Promega, Cat: G3582; Madison, WI, USA). Furthermore, cellular quantification of the seeded cells on the scaffolds was determined based on DNA staining, as previously described (Thermo Scientific™; Cat: 62249; Thermo Scientific; Waltham, MA, USA) [38]. The analysis of EV yield per cell was subsequently conducted, considering both metabolic activity and DNA content. Nuclear staining was conducted to qualitatively observe cellular adhesion on the nanofibrous scaffolds following the manufacturer’s instructions (Fisher, Cat: R37106; Waltham, MA, USA).

### 2.6. Statistical Analysis

Statistical analyses were conducted using GraphPad Prism 10 software. Two-way analysis of variance (ANOVA) with Tukey’s post hoc tests and normality assessments were applied to all datasets. Mechanical data and fibril diameter analysis underwent one-way ANOVA with Tukey’s post hoc test. Group comparisons were made at each timepoint, and temporal changes in EV secretion were quantitatively measured. Significance was defined as *p* < 0.05 (*), ** *p* < 0.01, *** *p* < 0.001, **** *p* < 0.0001. Data are expressed as mean ± standard deviation.

## 3. Results

### 3.1. Nanofibrous Scaffold Characterization

**Figure 1:**
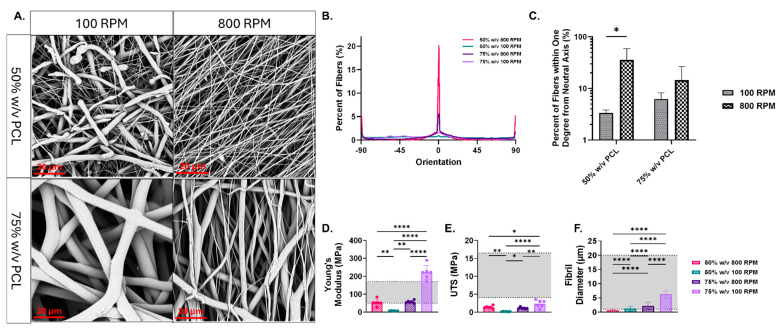
(**A**) SEM micrographs taken at 3000X magnification display the fibril morphology and configuration. (**B**) Fibril configuration was measured using ImageJ plugin, OrientationJ (*N* = 3 scaffolds per group). (**C**) The sum of fibers for each scaffold group within one degree from the neutral axis was measured and then plotted to demonstrate fibril orientation (*N* = 3 scaffolds per group). Mechanical testing employing a ramp-to-failure protocol with a strain rate of 0.3% per second was used to measure (**D**) Young’s modulus and (*N* = 6) (**E**) ultimate tensile strength (*N* = 6). (**F**) Fibril diameter was measured (*N* = 3 scaffolds) utilizing ImageJ. Mechanical and fibril diameter data are results of a one-way ANOVA with Tukey’s HSD post hoc test. Significance was defined as *p* < 0.05 (*), ** *p* < 0.01, **** *p* < 0.0001. Data are expressed as mean ± standard deviation. The gray range in panels (**D**–**F**) indicates range of Young’s Moduli (50–170 MPa), UTS (4.1–16.5 MPa) and fibril diameter (1–20 µm) for healthy human supraspinatus tendon [3,39].

SEM analysis demonstrated qualitatively that the nanofibrous PCL constructs had variation in alignment, with the scaffolds spun at 100 RPM having a more random configuration than the scaffolds spun at 800 RPM (Figure 1A). The fibers for each scaffold group within one degree from the neutral axis were quantified, summed, and plotted to illustrate fibril orientation (Figure 1B,C). We observed that the 50% solution spun at 800 RPM had significantly more fibers oriented about the neutral axis than when spun at 100 RPM, demonstrating enhanced alignment. A similar, though insignificant, trend was seen within the 75% polymer solution group. When comparing polymer densities within the same RPM, there were no significant variations in alignment. The scaffolds created from the polymer solutions with the higher concentration, 75% *w*/*v*, had significantly thicker fibril diameters than the lower concentrated solutions. Specifically, the group with a polymer concentration of 75% *w*/*v* at a mandrel speed of 100 RPM produced the thickest fibers, measuring 6.334 ± 1.356 µm. In contrast, the group with a polymer concentration of 50% *w*/*v* at a mandrel speed of 800 RPM yielded the thinnest fibers, with an average diameter of 0.6672 ± 0.2465 µm (Figure 1F). In our study, we investigated the mechanical properties of various scaffolds. We found that the scaffold with a concentration of 75% *w*/*v* and spun at 100 RPM demonstrated the highest Young’s Modulus (221.4 ± 39.26 MPa) and UTS (2.320 ± 1.041 MPa) compared to other scaffolds (Figure 1D,E). Notably, all scaffolds exhibited significantly different Young’s Moduli and UTS values, except for the 75% *w*/*v* concentration at 800 RPM and the 50% *w*/*v* concentration at 800 RPM, which showed similar Young’s Moduli (57.29 ± 7.671 MPa and 52.95 ± 18.11 MPa, respectively) and UTS (1.130 ± 0.2261 MPa and 1.379 ± 0.3615 MPa, respectively). All scaffolds had Young’s Moduli within the range of healthy human supraspinatus tendon (50–150 MPa) apart from the 50% *w*/*v* 100 RPM (7.151 ± 1.066 MPa) and the 75% *w*/*v* 100 RPM groups [39,40]. Considering the irregular fibril morphology, thinner fibril diameters, and lower stiffness observed, the scaffolds with 50% *w*/*v* concentration at 100 RPM most closely resemble pathological tendinous tissue [3,41,42].

### 3.2. Characterization of Fibroblast-EVs

**Figure 2:**
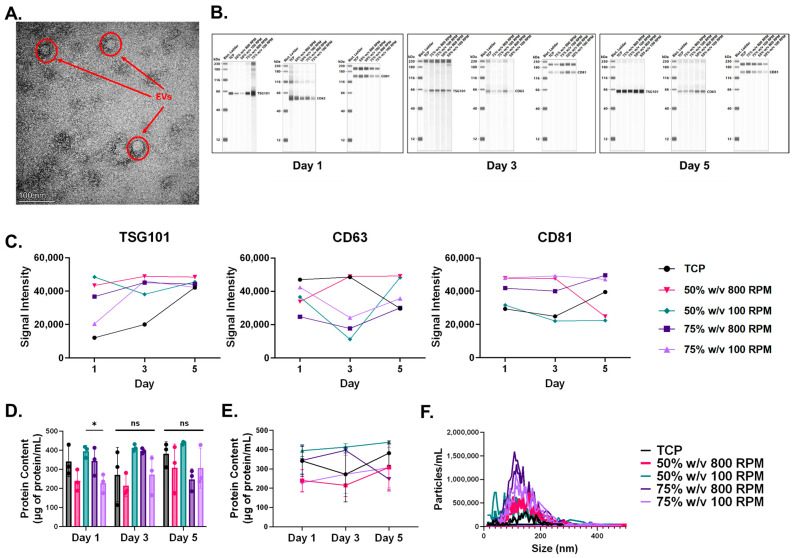
(**A**) TEM micrograph taken at 120 kX magnification displays spherical morphology of the EVs. (**B**) Western blot demonstrates expression of TSG101, CD63, and CD81 in all scaffold groups at all three timepoints. (**C**) Analysis of the Western blot bands indicate no significant variations in EV marker expressions. (**D**) BCA analysis quantifies the protein content in the EV samples, revealing significant variation between the 50% *w*/*v* and 75% *w*/*v* concentrations spun at 100 RPM on day 1. (**E**) BCA analysis demonstrates an overall consistency in EV protein content amongst the groups apart from the 50% *w*/*v* 100 RPM and 75% *w*/*v* 100 RPM groups on Day 1. (**F**) Particle size distribution of EVs amongst the scaffold groups. All data are results of a two-way ANOVA with Tukey’s HSD post hoc test. Significance was defined as *p* < 0.05 (*). Data are expressed as mean ± standard deviation.

TEM micrographs demonstrate round morphology of the isolated EVs (Figure 2A). Western blot analysis demonstrated consistent expression of exosome-specific markers TSG101, CD63, and CD81 across all experimental groups and timepoints (Figure 2B). TSG101 is an endosomal sorting complex required for transport (ESCRT)-I subunit implicated in endosome to cytosol release of biological cargo [43]. Furthermore, CD63 and CD81 are members of the tetraspanins family and are implicated in exosome biogenesis, cargo selection, targeting, and uptake [44]. No significant variations in EV marker expression were observed. Total protein content of the EVs was measured in all groups, with the only significant difference observed on Day 1 between the 50% *w*/*v* 100 RPM scaffolds and the 75% *w*/*v* 100 RPM scaffolds (Figure 2C). Protein content remained consistent amongst each group throughout the 5 day study (Figure 2D). NTA revealed size heterogeneity of the EVs, with average hydrodynamic diameters of 75 ± 16.36 nm, 69.17 ± 15.41 nm, 87.24 ± 21.60 nm, 71.16 ± 18.72 nm, and 66.37 ± 13.79 nm for the TCP, 50% *w*/*v* 800 RPM, 50% *w*/*v* 100 RPM, 75% *w*/*v* 800 RPM, and 75% *w*/*v* 100 RPM scaffolds, respectively, over the course of the five days (Figure 2E).

### 3.3. Assessment of Cellular Activity

**Figure 3:**
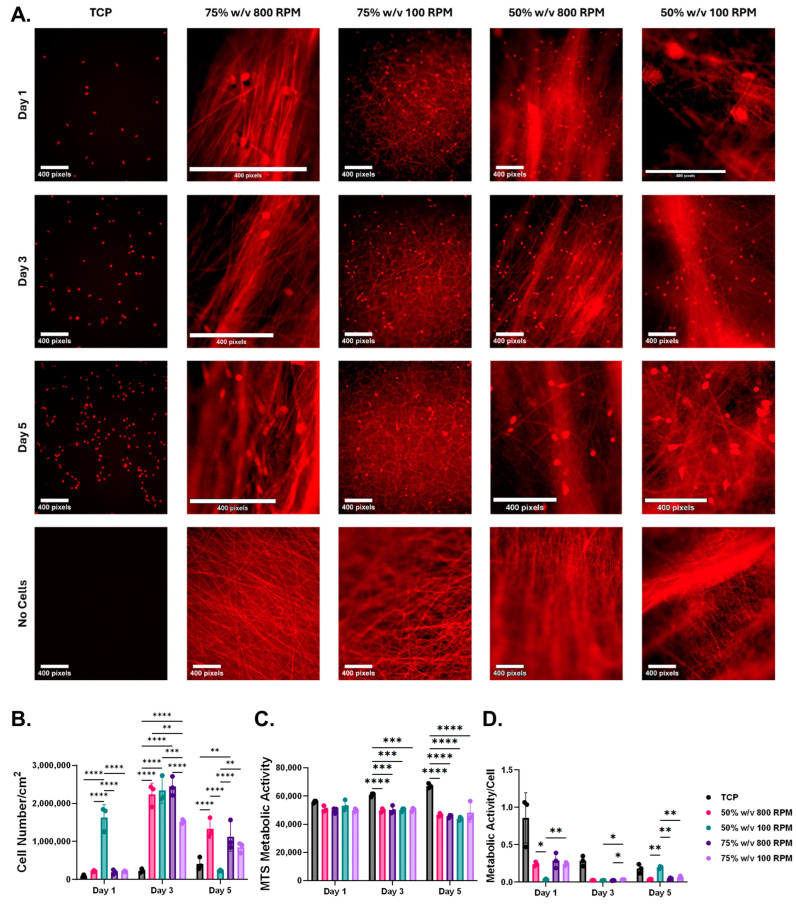
(**A**) Nuclear staining conducted to observe cell adhesion to nanofibrous scaffolds relative to TCP monolayer control at all timepoints. Fluorescence images were taken at 10X magnification. (**B**) Quantitative analysis of cellular proliferation demonstrates significantly greater number of cells in all nanofibrous scaffolds relative to TCP monolayer controls at Day 3. Day 5 demonstrates a similar trend except for the 50% *w*/*v* 100 RPM scaffold group (*N* = 3). (**C**) Metabolic activity of the cells was measured over the 5-day study, with the nanofibrous scaffolds having significantly reduced metabolic activity relative to the TCP monolayer control on Days 3 and 5 (*N* = 3). (**D**) Analysis of metabolic activity per cell demonstrates no significant variations between the nanofibrous scaffolds and the TCP monolayer control (*N* = 3). All data are results of a two-way ANOVA with Tukey’s HSD post hoc test. Significance was defined as *p* <  0.05 (*), ** *p* < 0.01, *** *p* < 0.001, **** *p* < 0.0001. Data are expressed as mean ± standard deviation.

Nuclear staining was performed to evaluate cellular adhesion on the nanofibrous scaffolds compared to the TCP controls (Figure 3A). The results demonstrated cellular adhesion embedded within the nanofibrous scaffolds. Notably, we observed increased cellular proliferation, qualitatively, in several nanofibrous scaffold groups relative to the TCP controls, specifically in the 75% *w*/*v* 100 RPM and 50% *w*/*v* 800 RPM groups on Days 1 and 3. Quantitative analysis of cellular proliferation showed that by Days 3 and 5, all nanofibrous scaffolds, except for the 50% *w*/*v* 100 RPM group on Day 5, exhibited a significant increase in cell number compared to the TCP monolayer controls (Figure 3B). Metabolic activity assessment revealed that all nanofibrous scaffolds had significantly reduced metabolic activity compared to the TCP monolayer controls on Days 3 and 5 (Figure 3C). To determine metabolic activity per cell, we normalized the metabolic activity to the cell number of each scaffold (Figure 3D). We found that all nanofibrous scaffolds exhibited significantly greater metabolic activity per cell compared to the 50% *w*/*v* 100 RPM group on Day 1. However, by Day 5, this trend reversed, with the 50% *w*/*v* 100 RPM group, emulating tendinopathic conditions, having significantly greater metabolic activity per cell over all other nanofibrous scaffolds. Importantly, there were no significant differences in metabolic activity per cell between the TCP monolayer cultures and the nanofibrous scaffolds.

### 3.4. Influence of Nanofibrous Scaffolds on EV Yield

**Figure 4:**
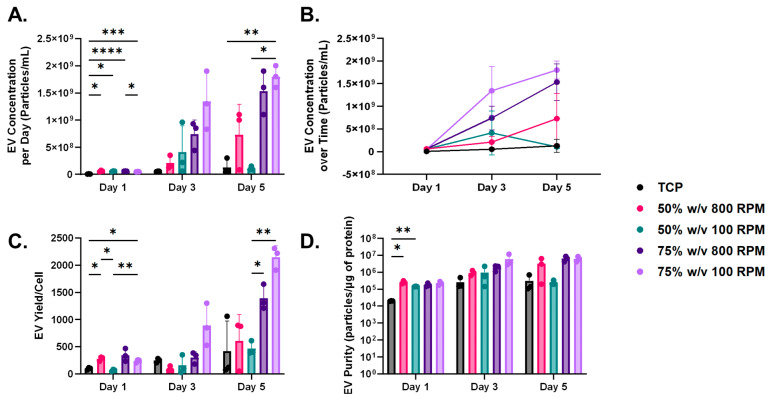
(**A**) Nanoparticle tracking analysis (NTA) revealed significantly greater extracellular vesicle (EV) secretion in all nanofibrous scaffolds compared to the tissue culture plastic (TCP) control on Day 1. This trend continued on Day 5, particularly in the 75% *w*/*v* 100 RPM group. Additionally, significant differences in EV secretion were observed on Day 5 between the 75% *w*/*v* and 50% *w*/*v* groups, both spun at 100 RPM. (**B**) A time-dependent increase in EV secretion was observed in the monolayer group between Days 1 and 3, and in the 75% *w*/*v* nanofibrous scaffolds between Days 1 and 5. (**C**) On Day 1, there was a significant increase in EV yield per cell in all groups compared to the monolayer control, except for the 50% *w*/*v* 100 RPM group. By Day 5, the 75% *w*/*v* group exhibited a significantly higher EV yield per cell compared to the 50% *w*/*v* 100 RPM group. (**D**) EV purity was assessed, with significant variations observed only on Day 1 between the 50% *w*/*v* 100 RPM group and the monolayer control. All data are results of a two-way ANOVA with Tukey’s HSD post hoc test. Significance was defined as *p* <  0.05 (*), ** *p* < 0.01, *** *p* < 0.001, **** *p* < 0.0001. Data are expressed as mean ± standard deviation.

NTA revealed that our nanofibrous scaffolds resulted in increased EV secretion (Figure 4A). Specifically, on Day 1, all nanofibrous scaffolds demonstrated significantly greater EV secretion than the TCP monolayer control. Additionally, the 75% *w*/*v* 100 RPM scaffolds exhibited significantly higher EV secretion compared to the 75% *w*/*v* 800 RPM scaffolds, indicating the impact of fibril alignment on EV secretion. By Day 5, the 75% *w*/*v* 100 RPM group also showed significantly more EVs than the 50% *w*/*v* 100 RPM scaffolds, highlighting the influence of polymer density on EV secretion. Additionally, we observed a time-dependent significant increase in EV secretion across all 75% *w*/*v* nanofibrous scaffold groups as well as our monolayer culture group (Figure 4B).

To assess EV yield per cell, we normalized the particle concentration to cell number. We observed that all nanofibrous scaffolds except for the 50% *w*/*v* 100 RPM group demonstrated significantly greater EV yield per cell. (Figure 4C). On Day 5, there was a significant increase in EV yield in the 75% *w*/*v* scaffold group relative to the 50% *w*/*v* 100 RPM nanofibrous scaffolds. Purity is defined as the ratio of particles to protein content as a measure of “true EVs” [45]. We found that EVs from nanofibrous scaffolds had greater purity than those from the TCP monolayer culture (Figure 4D). Specifically, on Day 1, the 50% *w*/*v* 100 RPM group showed significantly higher purity than the TCP controls.

## 4. Discussion

Many cellular components and processes involved in tendon repair remain poorly understood, highlighting the need for in vitro biomimetic models of healthy and diseased tendon tissues to better study the molecular mechanisms of cellular crosstalk in tendon healing, particularly through EVs. The objective of the current study was to observe cellular changes and EV biogenesis in microenvironments that are biomimetic of healthy and diseased tendinous tissue using in vitro biomaterial-based models. We hypothesized that nanofibrous scaffolds that are representative of pathologic tendinous tissue would yield enhanced EV secretion, increased cell proliferation, and metabolic activity. In its native state, healthy tendon exhibits a well-organized arrangement of collagen fibers that are thick, parallel, and tightly packed [46]. However, when subjected to injury or pathological conditions, the ECM of the tendon undergoes changes, resulting in thinner collagen fibers with greater variability in diameter [47]. This can also lead to impaired mechanics, with an increase in stiffness in fibrotic tendon conditions and reduced stiffness in the case of tendinitis and tendinosis [7,42]. Additionally, random configuration in fibril orientation is biomimetic of diseased tendinous tissue [48]. The current study also investigated the influence of mechanical properties of nanofibrous PCL constructs, fibril alignment, and diameter, on cellular activity and EV biogenesis. The only significant difference in fibril alignment was between the 50% *w*/*v* scaffolds spun at 800 RPM and 100 RPM. Scaffolds spun at 100 RPM had a random configuration, resembling diseased tendinous tissue, while the 50% *w*/*v* 800 RPM scaffolds had highly aligned fibrils. However, the 50% *w*/*v* at 800 RPM scaffold group had the thinnest fibril diameter and reduced stiffness, akin to pathologic tendon. Scaffold stiffness and fibril diameter are conjointly crucial to replicate healthy tendon, as these parameters are synergistically pertinent in maintaining tendinous structural integrity [46]. The 75% *w*/*v* scaffolds exhibited the highest Young’s Modulus and UTS, resembling healthy tendinous tissue due to their thicker fibrils and higher stiffness relative to the 50% *w*/*v* group.

Western blot analysis confirmed consistent expression of exosome-specific markers TSG101, CD63, and CD81 across all experimental groups and timepoints. Increased cellular proliferation was observed in several nanofibrous scaffold groups compared to the TCP controls. This phenomenon is consistent with many studies comparing two-dimensional cell cultures to 3D cell cultures, as the latter enables a greater surface area for cell growth and has proven to be more physiologically relevant over the former [49,50,51]. Nuclear staining also demonstrated cellular adhesion within the fibers of the nanofibrous scaffolds. As shown in Figure 3A, the 75% and 50% *w*/*v* scaffolds spun at 100 RPM, which more closely mimic fibrotic tendinous tissue, exhibit enhanced nuclear staining on days 1 and 3 compared to the TCP monolayer controls. These findings are consistent with Baldwin et al., which demonstrated enhanced cellular adhesion in randomly oriented nanofibrous scaffolds [52]. Additionally, Sooriyaarachchi et al. also demonstrated scaffolds with random nanofibers exhibited slightly higher cell proliferation compared to those with aligned fibers, a phenomenon observed in our nanofibrous scaffolds of random fibril configuration [53]. Surprisingly, all nanofibrous scaffolds exhibited significantly reduced cellular metabolic activity compared to the TCP monolayer controls, despite having significantly higher cell numbers as measured by nuclear staining proliferation analysis [54]. When normalized to cell proliferation data, no significant differences in metabolic activity per cell were observed between the TCP monolayer cultures and the nanofibrous scaffolds. This suggests that the discrepancy may be attributed to downregulated cellular metabolism upon reaching confluency, likely due to contact inhibition of cell proliferation [55,56].

Nanofibrous scaffolds significantly increased EV secretion compared to the TCP monolayer control on Day 1. This observation aligns with existing literature, which describes various methods for enhancing EV production, including the use of scaffolds and 3D cell culture systems [57]. Mesenchymal stem cells (MSCs) cultured on collagen scaffolds were found to secrete two times more EVs than those cultured on conventional monolayer culture systems [58]. Furthermore, the use of hollow fibers has been found to increase the production of EVs. Like nanofibrous scaffolds, hollow fibers possess a fibrous structure that provides a high surface area-to-volume ratio, facilitating the efficient mass transfer of EVs through the material. Gobin et al. demonstrated the use of bone marrow-derived MSC EVs in a hollow fiber bioreactor to enable a large amount of EVs to be derived from a large population of adherent cells [59]. Yet, there have been very limited studies on the use of nanofibrous, electrospun scaffolds for such applications, and to our knowledge, this is the first study to evaluate the structural and mechanical influence of electrospun scaffolds on EV secretion and biogenesis.

By Day 5, EV secretion was significantly higher in the 75% *w*/*v* 100 RPM scaffolds than in the monolayer group and the 50% *w*/*v* 100 RPM group, demonstrating the influence of both polymer density and fibril alignment. Higher-density polymer solutions tended to have increased mechanical stiffness. Additionally, our results demonstrated that nanofibrous scaffolds spun at higher RPMs had significantly greater stiffness than those spun at lower RPMs. This observation coincides with literature, as the alignment of the fibers affects stiffness, with the highest stiffness occurring when fibers are perfectly aligned [60]. The literature also indicates that lower-diameter fibers increase their crystallinity and molecular orientation, leading to enhanced mechanical strength and stiffness [61]. However, our results indicate otherwise, as the 75% *w*/*v* group overall had significantly thicker fibers, as well as being overall significantly stiffer than the 50% *w*/*v* group. This is most likely attributed to the high density of the polymeric solutions [62]. The likely reason the EV secretion from the cells seeded on the 75% *w*/*v* 100 RPM scaffolds was higher than cells seeded on the 50% *w*/*v* 100 RPM scaffolds is attributed to the significantly greater bulk mechanics of the 75% *w*/*v* scaffolds compared to the 50% *w*/*v* scaffolds [63].

Most nanofibrous scaffolds exhibited a higher EV yield per cell compared to the TCP control, with the 75% *w*/*v* scaffold group achieving the highest yield by Day 5. The 75% *w*/*v* nanofibrous scaffold group also had significantly greater EV secretion and yield per cell compared to the 50% *w*/*v* 100 RPM group. This observation not only demonstrates the influence of polymer density on EV biogenesis but also the bulk mechanics of the scaffolds. We postulate that the increased EV yield in the 75% *w*/*v* nanofibrous scaffold group is due to the higher stiffness of the scaffold. The 75% *w*/*v* group exhibited significantly greater mechanical properties compared to the 50% *w*/*v* 100 RPM scaffolds and demonstrated significantly higher EV secretion and yield per cell. Additionally, it is known that both cell sources and the microenvironment can influence the biological cargo of EVs, as well as their secretion and surface signals that dictate target tropism. Sneider et al. conducted experiments to elucidate the impact of matrix stiffness on EV biogenesis [64]. Cancer cells were cultured on in vitro matrices of human breast tumors (25 kPa) and softer normal tissue (0.5 kPa). They found that cells grown on the stiffer matrices exhibited heightened secretion of EVs compared to those on softer matrices. Additionally, they investigated the influence of substrate stiffness on EV cargo and biogenesis by isolating EVs from highly metastatic, triple-negative breast cancer cell lines cultured on both stiff and soft breast cancer tissue. Enhanced EV secretion was observed in the stiffer breast cancer tissues, and gene ontology analysis revealed enrichment of 55 proteins in EVs derived from the stiffer environments. This observed enrichment of proteins suggests that the mechanical properties of the stiffer environment may actively influence the composition and secretion of EVs, potentially affecting their ability to navigate through the ECM.

The existing literature indicates that EVs can passively traverse the ECM of tissues, although the typically small pore sizes of ECMs (<50 nm) may limit passive transport and diffusion, often necessitating reliance on the mechanical properties of the matrix [27]. EVs in stiff, stress-relaxing environments tend to escape ECM confinement more rapidly compared to those in softer, stress-relaxing microenvironments [27]. Studies using alginate-based hydrogels have shown that EV release is influenced by the mechanical properties of the hydrogel, with enhanced release observed from stiff, stress-relaxing hydrogels compared to stiff, elastic hydrogels [65]. A potential explanation for the enhanced EV secretion in our stiffer scaffold groups may be related to the enhanced ability of EVs to passively traverse through the scaffold. It has been suggested that aquaporin-1, a membrane protein that facilitates water transport, is present on the surface of EVs [65,66]. This protein facilitates the influx of water into EVs, thereby increasing their deformability and enhancing their capacity to navigate through confined environments such as ECM [65]. Additionally, mechanical stress can alter the activity of the ESCRT (endosomal sorting complex required for transport) pathway, leading to increased exosome secretion [67,68,69]. Various forms of cellular stress, including mechanical stress, can also activate autophagy, which in turn contributes to enhanced exosome release [70]. The enhanced EV secretion seen in these studies correlates with our findings that the 75% *w*/*v* group 100 RPM, the stiffest scaffold group, had the most EV secretion by Day 5.

We also assessed the purity of our EVs isolated from the monolayer and nanofibrous microenvironments. The concept of EV purity is pertinent as a highly purified EV population is desired, as protein impurities can affect the functional activity of EVs and therefore their cellular uptake and activity on other cells [57]. In our study, EVs from nanofibrous scaffolds emulating pathological tendinous microenvironments demonstrated higher purity compared to those from the TCP monolayer, particularly in the 50% *w*/*v* 100 RPM group on Day 1. Our results are consistent with findings that demonstrated that EVs isolated from in vitro 3D culture of umbilical derived MSCs in a bioreactor not only resulted in enhanced EV secretion but also EVs that had greater purity [71].

The 50% *w*/*v* nanofibrous scaffold group demonstrated the highest emulation of pathologic tendon conditions, characterized by thin fibril diameters and reduced mechanical properties. While there was no significant increase in EV secretion and yield per cell in the 50% *w*/*v* group compared to the 75% *w*/*v* group, cellular activity was notably affected by the pathological microenvironment. Specifically, in the 50% *w*/*v* 100 RPM group, a significant increase in metabolic activity per cell was observed by day 5 compared to the 75% *w*/*v* group. Additionally, day 3 showed enhanced cell proliferation in the 50% *w*/*v* group relative to the 75% *w*/*v* 100 RPM group.

It is emphasized that although previous studies have been conducted observing the influence of fibril diameter and orientation on fibroblasts, to our knowledge, this is the first study to observe such an influence on EV secretion and biogenesis. However, our study is not without limitations. Firstly, we did not assess EV cargo, which would be important for determining the influence of microenvironments on EV payload and function. This is significant because EVs encapsulate proteins, nucleic acids, metabolites, and miRNAs derived from the donor cell. Consequently, these cargo molecules can be associated with the cellular origin, providing valuable insights into the cellular and organ states, and facilitating molecular-level diagnosis of pathology [72]. Nevertheless, the bioactivity of EVs is determined not only by their internal content but also by their surface-associated molecules. These surface molecules influence the downstream signaling effects that EVs induce in target cells [73]. Our study demonstrated not only enhanced EV secretion but also a corresponding increase in cell proliferation compared to the monolayer control. Future work will involve incorporating omics’ approaches to analyze the molecular composition and functionality of the EVs. While our in vitro model aims to emulate both healthy and pathological tendon conditions, it would be more appropriate to use a physiologically relevant cell source such as tendon cells. However, the use of immortalized fibroblasts allowed us to optimize our tendon in vitro models. Furthermore, fibroblasts are the primary cell type in tendon and NIH3T3s exhibit characteristics similar to tendon fibroblasts [74,75]. Future research will address these limitations by incorporating physiologically relevant cell sources and assessing EV cargo to better understand the effects of the microenvironment on the EV payload. Additionally, we will assess cellular adhesion in our in vitro models using scanning electron microscopy (SEM) for a more detailed evaluation. This study focused exclusively on the biophysical changes occurring in the tendinous ECM during tendinopathy using a biomaterial model. Future research should incorporate collagen and immune cells into this model, as these factors are crucial in mediating matrix changes in tendinopathy.

## 5. Conclusions

In conclusion, this study explored the influence of biophysical alterations in the tendon healing microenvironment on cellular function, EV biogenesis, and EV profile, using biomaterials-based in vitro models of healthy and pathological tendinous tissue. Our findings confirmed that nanofibrous scaffolds, particularly those with higher polymer density and stiffness, significantly enhanced EV secretion and cell proliferation compared to TCP monolayer controls. The 75% *w*/*v* 100 RPM scaffolds, characterized by their higher Young’s Modulus and UTS, demonstrated the greatest EV yield and cellular activity, suggesting a pivotal role of scaffold mechanics in influencing these processes. Despite all nanofibrous scaffolds exhibiting reduced cellular metabolic activity relative to the TCP monolayers, the enhanced EV secretion and cell adhesion observed in these scaffolds underscore their potential in tissue engineering and regenerative medicine. Our study demonstrated that in response to stiffer microenvironments, there is an increase in EV secretion. This heightened EV release in stiffer microenvironments likely indicates a response to cellular stress [76]. In such conditions, cells often release more EVs as a means of communication, to remove unwanted substances or to modulate the immune response. 

Mechanosensing is a crucial mechanism in both cell–cell and cell-microenvironment interactions. It involves a cell’s response to mechanical forces, which can trigger the secretion of EVs for both local and distant signaling [69]. The mechanical properties of ECM have an outstanding impact on EV biogenesis. In MSC studies, it has been demonstrated that a rigid ECM can enhance EV secretion from tumor cells, thereby facilitating tumor growth [24,77]. EVs derived from 3D cultures contain higher levels of anti-inflammatory and anti-apoptotic factors compared to those from 2D cultures [78]. Future studies should therefore focus on assessing EV cargo to elucidate the impact of microenvironments on EV payload and employ physiologically relevant cell sources to further validate these findings. These efforts will deepen our understanding of the interplay between scaffold properties, cell behavior, and EV biogenesis, ultimately advancing the development of therapeutic strategies for tendon repair and regeneration.

## Data Availability

Data are contained within the article.
